# Vital sign predictors of severe influenza among children in an emergent care setting

**DOI:** 10.1371/journal.pone.0272029

**Published:** 2022-08-12

**Authors:** Suchitra Rao, Angela Moss, Molly Lamb, Bruce L. Innis, Edwin J. Asturias

**Affiliations:** 1 Department of Pediatrics (Infectious Diseases, Hospital Medicine and Epidemiology), University of Colorado School of Medicine and Children’s Hospital Colorado, Aurora, CO, United States of America; 2 Department of Pediatrics, University of Colorado School of Medicine and Children’s Hospital Colorado and Adult and Child Center for Health Outcomes Research and Delivery Science, Aurora, CO, United States of America; 3 Department of Epidemiology and Center for Global Health, University of Colorado School of Public Health, Aurora, CO, United States of America; 4 Center for Vaccine Innovation and Access, PATH, Seattle, WA, United States of America; 5 Department of Pediatrics, University of Colorado School of Medicine, Aurora, CO, United States of America; 6 Department of Epidemiology and Center for Global Health, Colorado School of Public Health, Aurora, CO, United States of America; PLOS, UNITED KINGDOM

## Abstract

**Background:**

Decisions regarding the evaluation of children with influenza infection rely on the likelihood of severe disease. The role of early vital signs as predictors of severe influenza infection in children is not well known. Our objectives were to determine the value of vital signs in predicting hospitalization/recurrent emergency department (ED) visits due to influenza infection in children.

**Methods:**

We conducted a prospective study of children aged 6 months to 8 years of age with influenza like illness evaluated at an ED/UC from 2016–2018. All children underwent influenza testing by PCR. We collected heart rate, respiratory rate and temperature, and converted heart rate (HR) and respiratory rate (RR) to z-scores by age. HR z scores were further adjusted for temperature. Our primary outcome was hospitalization/recurrent ED visits within 72 hours. Vital sign predictors with p< 0.2 and other clinical covariates were entered into a multivariable logistic regression model to determine odds ratios (OR) and 95% CI; model performance was assessed using the Brier score and discriminative ability with the C statistic.

**Results:**

Among 1478 children, 411 (27.8%) were positive for influenza, of which 42 (10.2%) were hospitalized or had a recurrent ED visit. In multivariable analyses, adjusting for age, high-risk medical condition and school/daycare attendance, higher adjusted respiratory rate (OR 2.09, 95%CI 1.21–3.61, p = 0.0085) was a significant predictor of influenza hospitalization/recurrent ED visits.

**Conclusions:**

Higher respiratory rate adjusted for age was the most useful vital sign predictor of severity among young children with PCR-confirmed influenza.

## Introduction

Influenza remains a significant public health threat, with unpredictable epidemics, pandemics and variable vaccine effectiveness leading to substantial yearly morbidity and mortality. While patients with certain medical conditions are at high risk for complications from influenza [1], severe illness can occur among healthy individuals, especially among children less than 5 years of age [2–4]. Early identification of children with influenza may lead to earlier treatment initiation and improved outcomes [5–7]). Determining early, objective measures that do not require laboratory or radiographic testing is of high value to help identify children at risk for higher morbidity and help guide providers’ clinical decision-making for enhanced care.

Despite advancements in diagnostics and therapeutics, one of the most significant challenges facing clinicians is in deciding which patients to test and treat for influenza. Clinically, influenza is often indistinguishable from other viruses, and there is no single symptom or sign with adequate sensitivity to make informed clinical decisions regarding testing or treatment [8, 9]. In one study, clinician judgment had sensitivity of only 29% in accurately diagnosing influenza [10]. While molecular testing platforms are more reliable than rapid antigen tests, they are expensive, and not widespread. Antivirals lead to a reduction in illness duration, and are associated with a decreased risk of lower respiratory tract infection, hospitalization and death [11, 12], but should be limited to patients who are at the highest risk for complications, to avoid widespread resistance to current therapeutics [13]. There is a critical need to increase the pre-test probability of children with influenza with the highest risk of morbidity, avoid excessive testing and treatment, and provide objective measures of severity to help determine when to escalate treatment or make decisions regarding disposition to improve the outcomes of children with severe disease.

Objective measures utilizing early vital sign data show promise in predicting more severe outcomes among adults with influenza using oxygen saturation, blood pressure, temperature and respiratory rate [14, 15]. However, pediatric evidence is sparse in the literature [16, 17]. Therefore, the objective of this analysis was to determine the usefulness of early vital signs in children to predict severe influenza infection defined as hospitalization or recurrent emergency department or urgent care visits.

## Methods

This study underwent full board review and was approved by the Colorado Multiple Institutions Review Board (COMIRB No.15-2308). This is a secondary analysis of a prospective study to evaluate a new moderate to severe classification of influenza in children [18]. Briefly, children 6 months-8 years of age presenting with influenza-like-illness (ILI) to the Children’s Hospital Colorado (CHCO) ED and an affiliated Urgent Care center were enrolled during two influenza seasons (January-April 2017 and November 2017-April 2018). ILI was defined as a temperature of ≥37.8⁰C and at least one of the following: cough, sore throat, runny nose or nasal congestion [19]. Children were excluded if they had respiratory symptom duration of greater than 14 days, if they were enrolled in the study within the prior 14 days, or if they had nurse-only visits. Nasopharyngeal swabs were obtained from all children and tested using the Cepheid Xpert® influenza real time RT- PCR (Sunnyvale, CA). Written informed consent was sought from all study participants. For children less than 7 years of age, written consent was obtained from parents/guardians, and for children 7 years of age and older, in addition to written consent from parents/guardians, additional written assent was obtained from children participating in the study per institutional policies. We evaluated our primary outcome firstly with the entire cohort, and secondly, with the subset of children who tested positive for influenza. Caregivers were interviewed in the ED or Urgent care regarding the child’s demographic characteristics, presenting symptoms, medical comorbidities, influenza vaccination status and household size. Vital sign data (heart rate, respiratory rate, oxygen saturation, blood pressure, capillary refill time) collected by chart abstraction, included the first set of vital signs and the highest heart rate/respiratory rate or temperature. Children were characterized as high-risk if they had a comorbidity increasing their risk of complications from influenza [20]. A vaccinated individual was defined as a child who received the adequate number of influenza vaccines for a given season, as defined by the Advisory Committee on Immunization Practices [21].

The primary outcome was hospitalization or recurrent ED or UC visits within 72 hours of the index visit. Data were summarized descriptively using frequencies for categorical variables and measures of central tendency for continuous variables. Proportions were compared using the Chi-square test or the Fisher’s exact test when needed. Mean values were compared using student’s t test. To examine the predictive value of vital sign data for PCR-confirmed influenza of subjects in the study cohort and hospitalization of the influenza-positive subjects, multivariable logistic regression was used. Heart rate and respiratory rate z score by age were calculated using a reference for expected heart rate and respiratory rate in hospitalized children [21]. Heart rate z scores were further adjusted for temperature [22]. A bivariable analysis was performed for each predictor of interest with outcome. Correlation between predictors was assessed with Pearson and Spearman correlation coefficients. Model performance was assessed with the scaled Brier score with higher values indicating better model performance. Discriminative ability was evaluated with the C statistic (Values >0.7 indicate good model discrimination) [23]. SAS v 9.4 (Cary, NC) was used for all analyses.

## Results

Among 1516 children with ILI enrolled in the study, 38 (2.5%) were excluded due to study withdrawal, meeting exclusion criteria or for other reasons. Of the remaining 1478 children, 252 were hospitalized, 45 had a recurrent ED visit within 72 hours of study enrollment; 411 (27.8%) tested positive for influenza type A or B, of which 28 (6.8%) were hospitalized and 14 (3.4%) had a recurrent ED visit within 72 hours of study enrollment. No hospitalized children who tested positive for influenza in our study required intensive care. We excluded 24 children who had a recurrent ED or UC visit after 72 hours from these analyses. The mean age of children with influenza was 4.2 years (IQR 2.2–6.1); 27% were considered at high-risk for influenza complications, and 37% were completely vaccinated against influenza for that season. Sociodemographic and clinical characteristics among children with influenza infection with and without hospitalization or recurrent ED visits are shown in Table 1. Children with influenza infection who were hospitalized or had a recurrent ED visit within 72 hours were more likely to have a high-risk medical condition (57% vs 23%, p < 0.01). The commonest reason for hospitalization among influenza positive patients was due to respiratory distress, hypoxia and dehydration. A higher proportion of children who were influenza negative were admitted for hypoxia compared with children who were influenza positive (3.8% vs 2.7%; p < 0.01).

**Table 1:** Sociodemographic characteristics of study participants.

Variables	Total	Influenza Positive	Influenza Negative	*p*-value^a^	Influenza Positive	Influenza Positive	*p*-value^a^
	(n = 1478)				Recurrent ED visit/Hospitalization	No Recurrent ED visit/Hospitalization	
	n (%)	(n = 411)	(n = 1067)				
		n (%)	n (%)		(n = 42)	(n = 363)	
					n (%)	n (%)	
**Age in years, mean (SD)**	3.2 (2.2)	4.2 (2.3)	2.9 (2.0)	**<0.01** ^**b**^	3.9 (2.2)	4.3 (2.4)	0.33^**b**^
**Male gender**	793 (54)	206 (50)	587 (55)	0.09	19 (45)	183 (50)	0.53
**Race/Ethnicity:**							
Hispanic/Latino	741 (50)	236 (57)	505 (47)	**<0.01**	24 (57)	210 (58)	0.93
White Non-Hispanic	477 (32)	108 (26)	369 (35)		12 (29)	93 (26)	
Black Non-Hispanic	127 (9)	38 (9)	89 (8)		4 (20)	34 (9)	
Other	133 (9)	29 (7)	104 (10)		2 (5)	26 (7)	
**High-risk medical condition**	425 (29)	111 (27)	314 (29)	0.36	24 (57)	85 (23)	**< .01**
**Insurance Status**:							
Private	450 (30)	100 (24)	350 (33)	**<0.01**	19 (21)	89 (25)	0.82
Medicaid	989 (67)	297 (72)	692 (65)		31 (74)	262 (72)	
Other	39 (3)	14 (3)	25 (2)		2 (5)	12 (3)	
**Vaccination Status:**							
Completely vaccinated	710 (50)	149 (37)	561 (54)	**<0.01**	19 (45)	127 (36)	0.52
Partially vaccinated	179 (13)	42 (11)	137 (13)		4 (10)	37 (11)	
Unvaccinated	542 (38)	207 (52)	335 (32)		19 (45)	186 (53)	
**Enrollment Location:**							
Urgent Care	476 (32)	143 (35)	333 (31)	0.19	14 (29)	129 (36)	0.40
ED	1,002 (68)	268 (65)	734 (69)		30 (71)	234 (64)	
**Attends daycare/school**	867 (59)	284 (69)	583 (55)	**<0.01**	24 (57)	258 (71)	0.06
**Test Result:**							
Influenza B	180 (44)	180 (44)	n/a	n/a	18 (43)	159 (44)	0.88
Influenza A	229 (56)	229 (56)	n/a	n/a	24 (57)	202 (56)	

a- Chi-square unless otherwise specified

b- b-Student’s T test

### Predictors of hospitalization or recurrent ED visit from influenza infection

The bivariable analyses of vital sign data as predictors of hospitalization among children with ILI and PCR-confirmed influenza illness are shown in Table 2. Temperature, heart rate, oxygen saturation and respiratory rate were significant predictors in bivariable analyses for the ILI cohort. Of these, clinically meaningful vitals sign data (peak heart rate, respiratory rate z score and initial oxygen) were used for the multivariable logistic regression models. The first model included vital signs only, and the second model included other covariates (age, high risk medical condition and school/daycare attendance) (S1 Table). Results of the multivariable analysis indicated respiratory rate z score (1.76 (95% CI 1.48–2.10) as a risk factor and high initial oxygen saturation (0.85 (95% CI 0.81–0.89) as a protective factor for hospitalization/recurrent ED visits among children with ILI. This model had improved discriminatory ability with the inclusion of age, high risk medical condition, and school/daycare attendance as additional covariates (c-index = 0.76) and performance (Scaled Brier score 0.17).

**Table 2:** Predictive value of vital sign data on hospitalization or recurrent ED visit within 72 hours among children with ILI and PCR-confirmed influenza evaluated in an ED and urgent care setting- bivariable analyses.

Variables	Total (n = 1454)	Hospitalized/ recurrent ED visit (n = 297)	Not hospitalized/ recurrent ED visit (n = 1157)	*p*-value	Influenza positive (n = 405) value (%)	Influenza positive hospitalized /recurrent ED visit (n = 42) value (%)	Influenza negative not Hospitalized/ recurrent ED visit (n = 363) value (%)	*p*-value^a^
Duration of fever (days), mean (SD)	2.8 (2.1)	3.0 (2.4)	2.7 (2.0)	0.43	2.9 (2.3)	3.9 (3.4)	2.7 (2.1)	0.06
Highest temperature in ED/UC, mean (SD)	38.4 (1.1)	38.6 (1.0)	38.3 (1.1)	< .01	38.6 (1.1)	38.9 (1.2)	38.6 (1.1)	0.19
Initial heart Rate, mean (SD)	143.5 (24.4)	151.5 (23.3)	141.5 (24.2)	< .01	138.3 (23.6)	144.3 (25.7)	137.6 (23.3)	0.08
Heart rate during highest temperature in ED/UC, mean (SD)	143.5 (24.4)	151.6 (23.2)	141.4 (24.1)	< .01	138.3 (23.4)	144.7 (24.8)	137.6 (23.1)	0.06
Peak heart rate z score (age/temp adjusted)	0.5 (0.9)	0.7 (1.0)	0.4 (0.9)	< .01	0.2 (0.9)	0.4 (0.9)	0.2 (0.9)	0.24
Initial heart rate z score (age/temp adjusted), mean (SD)	0.6 (1.0)	0.9 (1.0)	0.5 (0.9)	< .01	0.3 (0.9)	0.5 (1.0)	0.3 (0.9)	0.21
Initial oxygen Saturation, mean (SD)	95.3 (3.2)	93.3 (4.3)	95.8 (2.7)	< .01	95.9 (2.7)	94.6 (3.8)	96.0 (2.5)	< .01
Initial respiratory Rate, mean (SD)	35.3 (11.7)	42.5 (14.7)	33.4 (10.0)	< .01	31.2 (9.4)	36.1 (13.2)	30.7 (8.7)	< .01
Initial respiratory rate z score (age adjusted), mean (SD)	0.8 (0.9)	1.3 (0.9)	0.7 (0.8)	< .01	0.6 (0.8)	1.1 (0.8)	0.6 (0.7)	< .01

ED—Emergency Department, UC—Urgent Care, SD—standard deviation

a—Student’s T test

**Fig 1:**
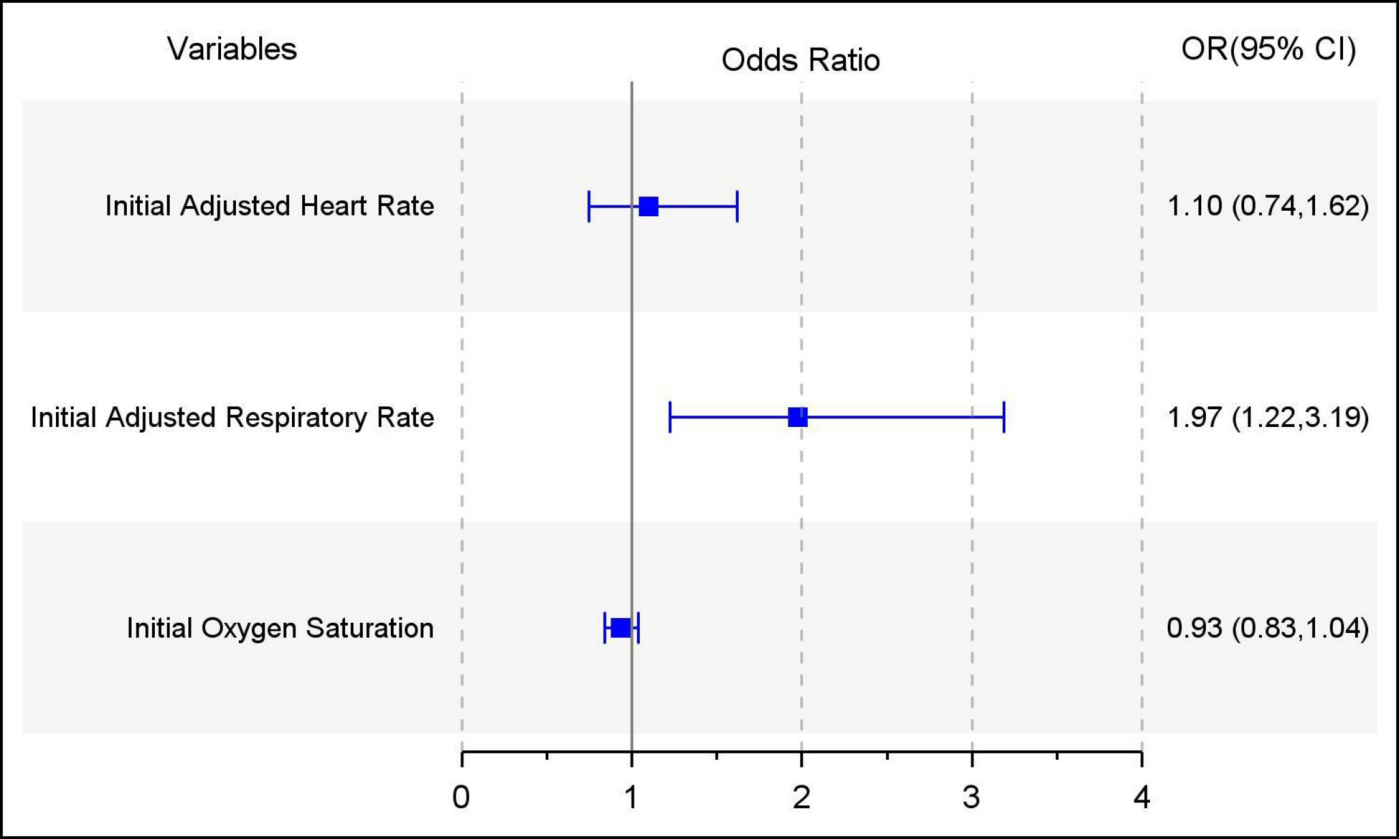
Odds of hospitalization and recurrent ED visits among children with PCR-confirmed influenza using vital sign data as clinical predictors, model adjusted for age, high risk co-morbidities, school or daycare attendance.

Initial oxygen saturation and initial respiratory rate were significant predictors for severe influenza infection in bivariable analyses and were subsequently used for the multivariable logistic regression. In multivariable analyses, among children with influenza infection, only higher adjusted respiratory rate z score remained a significant predictor of hospitalization or recurrent ED visits (OR 1.84, 95%CI 1.17–2.90) (Fig 1). However, the model had poor discrimination (c-index = 0.67) and performance (Scaled Brier score = 0.05). For improved model performance, we conducted analyses using a second model that adjusted for age, high risk co-morbidities and school or daycare attendance. Initial higher adjusted respiratory rate remained a significant predictor of hospitalization or recurrent ED visits (OR 1.97 1.22–3.19), with overall improved discrimination (c-index 0.77) and performance (Scaled Brier score 0.12) (Fig 1).

## Discussion

Our observational cohort study of children with influenza like illness being evaluated in the ED/UC setting demonstrated that higher respiratory rate adjusted for age was the most significant vital sign predictor of hospitalization or recurrent ED/UC visits within 72 hours among young children with PCR-confirmed influenza. While adjusted heart rate during peak temperature and lower oxygen saturation were significant in bivariable analyses, they were no longer significant in our adjusted analyses. Model performance improved significantly after including age, high risk co-morbidities and school or daycare attendance. For children with ILI, the most important vital signs predicting hospitalization among children with ILI were both age adjusted respiratory rate, and oxygen saturation. Our findings demonstrate that a predictive model which includes age, respiratory rate z score, high risk co-morbidities and school or daycare attendance can help risk-stratify children with more severe outcomes from influenza infection.

Early vital sign data predicting more severe outcomes among children with influenza is limited in the literature. One matched case-control study among outpatients with respiratory symptoms showed that fever was an independent predictor for influenza, however, the study did not investigate other vital sign measurements nor its applicability to hospitalization and severity [16]. Another study of hospitalized children reported that low initial oxygen saturation at admission predicted the need for intensive care [17], but this was not found to be an independent predictor in multivariate analysis. Low oxygen saturation has also been shown to be a useful predictor of severe outcomes in a respiratory index of severity in children (RISC) score, which forecasts the probability of death in a young child with lower respiratory tract infection [24].

Studies of influenza infection in children and adults have similarly demonstrated the value of respiratory rate in predicting hospitalization. One study evaluating vital signs as predictors of hospitalization of children and adults with H1N1 influenza, demonstrated that in multivariate regression analyses of all vital signs, tachypnea was a significant risk factor for hospital admission (OR = 1.1; 95% CI 1.02 to 1.13, p<0.01) [25]. Our findings are also consistent with another study evaluating adults with H1N1 influenza demonstrating that tachypnea is a significant risk factor for hospitalization [26].

Respiratory rate has been shown to be a useful predictor of severity for acute respiratory infections and pneumonia. For example, children with tachypnea as defined by the World Health Organization (WHO) respiratory rate thresholds are more likely to have pneumonia than children without tachypnea [27]. However, using tachypnea as a dichotomous variable may decrease the overall model performance. While using vital sign z scores rather than the presence or absence of tachypnea is more complex, it enhances the statistical power over using a dichotomous threshold, and these data can be effectively used in EHR systems and computer algorithms to risk-stratify children [28, 29]. The utility of this approach has been shown in model predictors for identifying children with serious bacterial infections, showing that the most robust model used age adjusted heart rate and respiratory rates [30].

Other studies have also demonstrated that hypoxia or requirement for oxygen was also an important risk factor for hospitalization or ICU admission [31, 32], which we found in our bivariable analyses for influenza-confirmed infection, but after adjusting for other important covariates, this was no longer significant. Our study suggests that respiratory rate is a more reliable predictor of influenza severity than oxygen saturation in children, but our findings may be difficult to interpret since the effects of higher altitude in Colorado may have impacted the interpretation of oxygen saturation in this study [33–35]. Further, the higher proportion of hypoxia among children testing negative for influenza likely represented younger children with RSV infection, which was co-circulating during the enrollment period of our study, known to be associated with hypoxia in children [36]. The stronger association of hypoxia due to RSV may also explain why oxygen saturation was a significant predictor for our ILI cohort, but not for our influenza-confirmed cohort.

Decisions regarding the investigation and treatment of children with influenza and infection rely on factors such as incidence of influenza in the community and likelihood of severe disease. Early predictors can help the clinician target testing and treatment to high-risk individuals, which is especially crucial during times of limited testing capacity, as evidenced by the current pandemic. Existing respiratory severity assessment scores may underestimate the risk of influenza severity, especially in younger individuals [37], and therefore a model specific to influenza is necessary. A predictive model using objective early clinical parameters can be incorporated in the clinical setting through clinical decision support tools in the EHR, for risk stratification for influenza infection, which can help to standardize care, while reducing unnecessary testing and antiviral use. The ED and inpatient floors are potential settings in which such clinical tools can have a high impact, since the population of interest is sicker, reliable testing platforms are available, thus enhancing the diagnosis, prompt initiation of antivirals and ongoing monitoring among those with confirmed influenza infection [38]. Such clinical guidance would be especially important when resources are limited, as evidenced during the COVID-19 pandemic, to help the provider triage the appropriate level of care and determine appropriate therapies, while conserving resources.

There are several limitations that warrant discussion. First, our study was conducted at a single center among children evaluated in an ED or UC setting, which may limit its external validity to other sites and settings. Extrapolation of our clinical prediction tool to other settings is underway. Our cohort of children with influenza had a low rate of hospitalization, so we used a composite outcome of hospitalization or recurrent visits, but limited the recurrent visits to within 72 hours of the index hospitalization. Given the rare outcome, the model was sensitive to overfitting, but when comparing our logistic regression model with 3 covariates compared with 6 covariates, there was little change in the 95% confidence intervals, indicating stability of our expanded model, which is considered an acceptable analytic approach [39, 40]. Next, we used vital sign data collected during the index visit and were not able to account for day of illness in our model, and thus our vital sign data was collected during different phases of the illness course, when they sought care. Our study was conducted in a setting that used machine-read heart rate and respiratory rate data, but these have been shown to correlate well with electrocardiograph heart rate and physician measures [41, 42]. Finally, we did not explore other predictive modeling approaches, such as including influenza results as a covariate in the ILI model, but will be the subject of future study.

Our study demonstrates the clinical utility of a prediction model that incorporates age, high risk medical condition, school or daycare attendance and respiratory rate z score in predicting hospitalization or recurrent ED visits for children aged 6 months to 8 years of age with influenza infection. This study has important implications for researchers as well as clinicians, because determining early, objective measures that do not require laboratory or radiographic testing is of high value to help improve the pre-test probability for determining which children are at risk for higher morbidity, to help guide providers’ clinical decision-making process regarding testing and treatment. These findings are especially timely during a time when influenza is co-circulating with COVID-19, when there may be shortages in testing reagents, trained personnel and more restrictive testing capabilities, highlighting an important need to identify which children should be tested for influenza as well as SARS-CoV-2. Further work including z score thresholds and external validation is ongoing, but these findings show promise for use in clinical prediction tools in the ED and hospital setting.

## Supporting information

S1 Table Multivariable logistic regression analyses evaluating association between vital sign data and hospitalization/recurrent ED visits among children with ILI. (DOCX)

## Abbreviations

ED: Emergency Department
UC: Urgent Care
ILI: influenza like illness
CHCO: Children’s Hospital Colorado
OR: Odds Ratio
IQR: Interquartile range

## References

[1] Grohskopf LA, Alyanak E, Broder KR, Walter EB, Fry AM, Jernigan DB. Prevention and Control of Seasonal Influenza with Vaccines: Recommendations of the Advisory Committee on Immunization Practices—United States, 2019–20 Influenza Season. MMWR Recomm Rep. 2019;68(3):1–21. doi: 10.15585/mmwr.rr6803a1 31441906 PMC6713402

[2] Thompson WW, Shay DK, Weintraub E, Brammer L, Bridges CB, Cox NJ, et al. Influenza-associated hospitalizations in the United States. Jama. 2004;292(11):1333–40. doi: 10.1001/jama.292.11.1333 15367555

[3] Zhou H, Thompson WW, Viboud CG, Ringholz CM, Cheng PY, Steiner C, et al. Hospitalizations associated with influenza and respiratory syncytial virus in the United States, 1993–2008. Clinical infectious diseases: an official publication of the Infectious Diseases Society of America. 2012;54(10):1427–36. 10.1093/cid/cis211 PMC3334364 22495079

[4] Dawood FS, Fiore A, Kamimoto L, Nowell M, Reingold A, Gershman K, et al. Influenza-associated pneumonia in children hospitalized with laboratory-confirmed influenza, 2003–2008. The Pediatric infectious disease journal. 2010;29(7):585–90. doi: 10.1097/inf.0b013e3181d411c5 20589966 PMC5856105

[5] Coffin SE, Leckerman K, Keren R, Hall M, Localio R, Zaoutis TE. Oseltamivir shortens hospital stays of critically ill children hospitalized with seasonal influenza: a retrospective cohort study. The Pediatric infectious disease journal. 2011;30(11):962–6. doi: 10.1097/INF.0b013e318232ede9 21997661 PMC3426912

[6] Launes C, Garcia-Garcia JJ, Jordan I, Martinez-Planas A, Selva L, Munoz-Almagro C. 2009 Influenza A H1N1 infections: delays in starting treatment with oseltamivir were associated with a more severe disease. Pediatr Infect Dis J. 2011;30(7):622–5. doi: 10.1097/INF.0b013e3182093397 21200359

[7] Eriksson CO, Graham DA, Uyeki TM, Randolph AG. Risk factors for mechanical ventilation in U.S. children hospitalized with seasonal influenza and 2009 pandemic influenza A*. Pediatr Crit Care Med. 2012;13(6):625–31. doi: 10.1097/PCC.0b013e318260114e 22895006 PMC6615726

[8] Call SA, Vollenweider MA, Hornung CA, Simel DL, McKinney WP. Does this patient have influenza? Jama. 2005;293(8):987–97. doi: 10.1001/jama.293.8.987 15728170

[9] Ebell MH, White LL, Casault T. A systematic review of the history and physical examination to diagnose influenza. J Am Board Fam Pract. 2004;17(1):1–5. doi: 10.3122/jabfm.17.1.1 15014046

[10] Stein J, Louie J, Flanders S, Maselli J, Hacker JK, Drew WL, et al. Performance characteristics of clinical diagnosis, a clinical decision rule, and a rapid influenza test in the detection of influenza infection in a community sample of adults. Ann Emerg Med. 2005;46(5):412–9. doi: 10.1016/j.annemergmed.2005.05.020 16271670

[11] Hsu J, Santesso N, Mustafa R, Brozek J, Chen YL, Hopkins JP, et al. Antivirals for treatment of influenza: a systematic review and meta-analysis of observational studies. Ann Intern Med. 2012;156(7):512–24. doi: 10.7326/0003-4819-156-7-201204030-00411 22371849 PMC6679687

[12] Louie JK, Yang S, Samuel MC, Uyeki TM, Schechter R. Neuraminidase inhibitors for critically ill children with influenza. Pediatrics. 2013;132(6):e1539–45. doi: 10.1542/peds.2013-2149 24276847 PMC6637754

[13] Ison MG. Optimizing antiviral therapy for influenza: understanding the evidence. Expert Rev Anti Infect Ther. 2015;13(4):417–25. doi: 10.1586/14787210.2015.1018183 25695406

[14] Singanayagam A, Singanayagam A, Wood V, Chalmers JD. Factors associated with severe illness in pandemic 2009 influenza a (H1N1) infection: implications for triage in primary and secondary care. The Journal of infection. 2011;63(4):243–51. doi: 10.1016/j.jinf.2011.07.014 21839111

[15] Perez-Padilla R, de la Rosa-Zamboni D, Ponce de Leon S, Hernandez M, Quinones-Falconi F, Bautista E, et al. Pneumonia and respiratory failure from swine-origin influenza A (H1N1) in Mexico. The New England journal of medicine. 2009;361(7):680–9. doi: 10.1056/NEJMoa0904252 19564631

[16] Heinonen S, Peltola V, Silvennoinen H, Vahlberg T, Heikkinen T. Signs and symptoms predicting influenza in children: a matched case-control analysis of prospectively collected clinical data. European journal of clinical microbiology & infectious diseases: official publication of the European Society of Clinical Microbiology. 2012;31(7):1569–74. 10.1007/s10096-011-1479-4 22080425

[17] Ma HY, Wu JL, Lu CY, Chen JM, Lee PI, Chang LY, et al. Risk factors associated with severe influenza virus infections in hospitalized children during the 2013 to 2014 season. Journal of microbiology, immunology, and infection = Wei mian yu gan ran za zhi. 2015. doi: 10.1016/j.jmii.2015.05.015 26216185

[18] Rao S YE, Moss A, Lamb MM, Schuind A, Bekkat-Berkani B, Innis BL, et al. Evaluation of a new Clinical Endpoint for Moderate-to-severe Influenza Disease in Children: A Prospective Cohort Study. Journal of the Pediatric Infectious Diseases Society (in press). 2019;(in press). 10.1093/jpids/piz075 PMC7495912 31724050

[19] Budd ABL, Grohskopf L, Campbell A, Dugan V, Wentworth DE, Brammer L. Manual for the Surveillance of Vaccine-Preventable Diseases.

[20] Grohskopf LA, Sokolow LZ, Broder KR, Walter EB, Bresee JS, Fry AM, et al. Prevention and Control of Seasonal Influenza with Vaccines: Recommendations of the Advisory Committee on Immunization Practices—United States, 2017–18 Influenza Season. MMWR Recomm Rep. 2017;66(2):1–20. doi: 10.15585/mmwr.rr6602a1 28841201 PMC5837399

[21] Bonafide CP, Brady PW, Keren R, Conway PH, Marsolo K, Daymont C. Development of heart and respiratory rate percentile curves for hospitalized children. Pediatrics. 2013;131(4):e1150–7. doi: 10.1542/peds.2012-2443 23478871 PMC4074640

[22] Daymont C, Bonafide CP, Brady PW. Heart rates in hospitalized children by age and body temperature. Pediatrics. 2015;135(5):e1173–81. doi: 10.1542/peds.2014-3738 25917984 PMC4411783

[23] Steyerberg EW, Vickers AJ, Cook NR, Gerds T, Gonen M, Obuchowski N, et al. Assessing the performance of prediction models: a framework for traditional and novel measures. Epidemiology. 2010;21(1):128–38. doi: 10.1097/EDE.0b013e3181c30fb2 20010215 PMC3575184

[24] Reed C, Madhi SA, Klugman KP, Kuwanda L, Ortiz JR, Finelli L, et al. Development of the Respiratory Index of Severity in Children (RISC) score among young children with respiratory infections in South Africa. PloS one. 2012;7(1):e27793. doi: 10.1371/journal.pone.0027793 22238570 PMC3251620

[25] Alshahrani M, Alsubaie A, Alshamsy A, Alkhliwi B, Alshammari H, Alshammari M, et al. Can the emergency department triage category and clinical presentation predict hospitalization of H1N1 patients? Open Access Emerg Med. 2019;11:221–8. doi: 10.2147/OAEM.S204110 31572026 PMC6757191

[26] Echevarria-Zuno S, Mejia-Arangure JM, Mar-Obeso AJ, Grajales-Muniz C, Robles-Perez E, Gonzalez-Leon M, et al. Infection and death from influenza A H1N1 virus in Mexico: a retrospective analysis. Lancet. 2009;374(9707):2072–9. doi: 10.1016/S0140-6736(09)61638-X 19913290

[27] Palafox M, Guiscafre H, Reyes H, Munoz O, Martinez H. Diagnostic value of tachypnoea in pneumonia defined radiologically. Arch Dis Child. 2000;82(1):41–5. doi: 10.1136/adc.82.1.41 10630911 PMC1718193

[28] Royston P, Altman DG, Sauerbrei W. Dichotomizing continuous predictors in multiple regression: a bad idea. Stat Med. 2006;25(1):127–41. doi: 10.1002/sim.2331 16217841

[29] Naggara O, Raymond J, Guilbert F, Roy D, Weill A, Altman DG. Analysis by categorizing or dichotomizing continuous variables is inadvisable: an example from the natural history of unruptured aneurysms. AJNR Am J Neuroradiol. 2011;32(3):437–40. doi: 10.3174/ajnr.A2425 21330400 PMC8013096

[30] Spruijt B, Vergouwe Y, Nijman RG, Thompson M, Oostenbrink R. Vital signs should be maintained as continuous variables when predicting bacterial infections in febrile children. J Clin Epidemiol. 2013;66(4):453–7. doi: 10.1016/j.jclinepi.2012.09.014 23306061

[31] Vasoo S, Singh K, Trenholme GM. Predicting need for hospitalization of patients with pandemic (H1N1) 2009, Chicago, Illinois, USA. Emerg Infect Dis. 2010;16(10):1594–7. doi: 10.3201/eid1610.091889 20875287 PMC3294388

[32] Dalziel SR, Thompson JM, Macias CG, Fernandes RM, Johnson DW, Waisman Y, et al. Predictors of severe H1N1 infection in children presenting within Pediatric Emergency Research Networks (PERN): retrospective case-control study. BMJ. 2013;347:f4836. doi: 10.1136/bmj.f4836 23940290 PMC3741086

[33] Crocker ME, Hossen S, Goodman D, Simkovich SM, Kirby M, Thompson LM, et al. Effects of high altitude on respiratory rate and oxygen saturation reference values in healthy infants and children younger than 2 years in four countries: a cross-sectional study. Lancet Glob Health. 2020;8(3):e362–e73. doi: 10.1016/S2214-109X(19)30543-1 32087173 PMC7034060

[34] Yaron M, Niermeyer S, Lindgren KN, Honigman B, Strain JD, Cairns CB. Physiologic response to moderate altitude exposure among infants and young children. High Alt Med Biol. 2003;4(1):53–9. doi: 10.1089/152702903321488988 12713712

[35] Ucros S, Granados CM, Castro-Rodriguez JA, Hill CM. Oxygen Saturation in Childhood at High Altitude: A Systematic Review. High Alt Med Biol. 2020;21(2):114–25. doi: 10.1089/ham.2019.0077 32239983

[36] Darville T, Yamauchi T. Respiratory syncytial virus. Pediatr Rev. 1998;19(2):55–61. doi: 10.1542/pir.19-2-55 9473944

[37] Pereira JM, Moreno RP, Matos R, Rhodes A, Martin-Loeches I, Cecconi M, et al. Severity assessment tools in ICU patients with 2009 influenza A (H1N1) pneumonia. Clin Microbiol Infect. 2012;18(10):1040–8. doi: 10.1111/j.1469-0691.2011.03736.x 22264290

[38] Kappen TH, van Loon K, Kappen MA, van Wolfswinkel L, Vergouwe Y, van Klei WA, et al. Barriers and facilitators perceived by physicians when using prediction models in practice. Journal of clinical epidemiology. 2016;70:136–45. doi: 10.1016/j.jclinepi.2015.09.008 26399905

[39] van Smeden M, de Groot JA, Moons KG, Collins GS, Altman DG, Eijkemans MJ, et al. No rationale for 1 variable per 10 events criterion for binary logistic regression analysis. BMC Med Res Methodol. 2016;16(1):163. doi: 10.1186/s12874-016-0267-3 27881078 PMC5122171

[40] Vittinghoff E, McCulloch CE. Relaxing the rule of ten events per variable in logistic and Cox regression. Am J Epidemiol. 2007;165(6):710–8. doi: 10.1093/aje/kwk052 17182981

[41] Iyriboz Y, Powers S, Morrow J, Ayers D, Landry G. Accuracy of pulse oximeters in estimating heart rate at rest and during exercise. Br J Sports Med. 1991;25(3):162–4. doi: 10.1136/bjsm.25.3.162 1777787 PMC1478836

[42] Alwadhi V, Sarin E, Kumar P, Saboth P, Khera A, Gupta S, et al. Measuring accuracy of plethysmography based respiratory rate measurement using pulse oximeter at a tertiary hospital in India. Pneumonia (Nathan). 2020;12:4. doi: 10.1186/s41479-020-00067-2 32518740 PMC7273681

