# Supplementary material for: Vital sign predictors of severe influenza among children in an emergent care setting
Source: PLoS One. 2022 Aug 12;17(8):e0272029. doi: 10.1371/journal.pone.0272029 (PMC9374253; doi:10.1371/journal.pone.0272029)
Supplement: S1 Table — (DOCX) [file pone.0272029.s001.docx]

**S1 Table.** **Multivariable logistic regression analyses evaluating association between vital sign data and hospitalization/recurrent ED visits among children with ILI**

|  | **Model 1** | | **Model 2** | |
| --- | --- | --- | --- | --- |
| **Variable** | **Adjusted Odds Ratio (95%CI)** | ***p* value** | **Adjusted Odds Ratio (95%CI)** | ***p* value** |
| Initial Heart Rate Z Score (adjusted for age and temp) | 1.14 (0.98,1.33) | 0.0836 | 1.10(0.94,1.29) | 0.2269 |
| Initial Respiratory Rate Z Score (adjusted for age) | 1.76 (1.48,2.10) | <0.0001 | 1.69 (1.42,2.02) | <0.0001 |
| Initial oxygen saturation | 0.85 (0.81,0.89) | <0.0001 | 0.85 (0.81,0.89) | <0.0001 |
| Age in years |  |  | 0.87(0.81,0.95) | 0.0013 |
| High risk medical condition: Yes vs No |  |  | 2.32 (1.72,3.12) | <0.0001 |
| Attends school/daycare: Yes vs No |  |  | 1.20 (0.87,1.65) | 0.2650 |
| c-index | 0.73 | | 0.76 | |
| Brier Score | 0.139 | | 0.135 | |
| Scaled Brier Score | 15.3% | | 17.34% | |
